# Bacterial Cellulose: Production, Characterization, and Application as Antimicrobial Agent

**DOI:** 10.3390/ijms222312984

**Published:** 2021-11-30

**Authors:** Dibyajit Lahiri, Moupriya Nag, Bandita Dutta, Ankita Dey, Tanmay Sarkar, Siddhartha Pati, Hisham Atan Edinur, Zulhisyam Abdul Kari, Noor Haslina Mohd Noor, Rina Rani Ray

**Affiliations:** 1 Department of Biotechnology, University of Engineering & Management, Kolkata 700156, West Bengal, India; dibyajit.lahiri@uem.edu.in (D.L.); moupriya.nag@uem.edu.in (M.N.); 2 Department of Biotechnology, Maulana Abul Kalam Azad University of Technology, Haringhata 700156, West Bengal, India; bandita2611@gmail.com; 3 Department of Pathology, Belle Vue Clinic, Kolkata 700156, West Bengal, India; ankita.dey16061996@gmail.com; 4 Malda Polytechnic, West Bengal State Council of Technical Education, Government of West Bengal, Malda 732102, West Bengal, India; tanmays468@gmail.com; 5 Skills Innovation & Academic Network (SIAN) Institute, Balasore 756001, Odisha, India; patisiddhartha@gmail.com; 6 NatNov Bioscience Private Limited, Balasore 756001, Odisha, India; 7 School of Health Sciences, Universiti Sains Malaysia, Health Campus, Kubang Kerian 16150, Kelantan, Malaysia; edinur@usm.my; 8 Faculty of Agro Based Industry, Universiti Malaysia Kelantan, Jeli 17600, Kelantan, Malaysia; zulhisyam.a@umk.edu.my; 9 Haematology Department, School of Medical Sciences, Universiti Sains Malaysia, Health Campus, Kubang Kerian 16150, Kelantan, Malaysia

**Keywords:** natural polymer, biocomposite, nanocomposite, antimicrobial, antibiofilm

## Abstract

Bacterial cellulose (BC) is recognized as a multifaceted, versatile biomaterial with abundant applications. Groups of microorganisms such as bacteria are accountable for BC synthesis through static or agitated fermentation processes in the presence of competent media. In comparison to static cultivation, agitated cultivation provides the maximum yield of the BC. A pure cellulose BC can positively interact with hydrophilic or hydrophobic biopolymers while being used in the biomedical domain. From the last two decades, the reinforcement of biopolymer-based biocomposites and its applicability with BC have increased in the research field. The harmony of hydrophobic biopolymers can be reduced due to the high moisture content of BC in comparison to hydrophilic biopolymers. Mechanical properties are the important parameters not only in producing green composite but also in dealing with tissue engineering, medical implants, and biofilm. The wide requisition of BC in medical as well as industrial fields has warranted the scaling up of the production of BC with added economy. This review provides a detailed overview of the production and properties of BC and several parameters affecting the production of BC and its biocomposites, elucidating their antimicrobial and antibiofilm efficacy with an insight to highlight their therapeutic potential.

## 1. Introduction

Cellulose is a prime biopolymer due to its extensive productive importance. Chemically, it is linear homopolysaccharide, composed of β-D-glucopyranose units that remain linked by β-1,4 glycosidic bonds. Apart from being the most important structural component of the primary cell wall of green plants, cellulose is found to also be present in bacteria [1,2,3]. Cellulose is a group of carbohydrates that contains considerable amounts of hydroxyl groups that remain existent in the form of polymer chain [4] and has wide applications in pulp and paper and pharmaceutical industries and as a renewable fuel source. However, the plant cellulose is often associated with lignin, pectin, hemicellulose, and other biogenic products, which has made it difficult to obtain pure cellulose as substrate [5,6].

Microbial cellulose is considered as a source of pure cellulose, which is usually synthesized by the bacteria. Because of its high purity and distinct physicochemical characteristics, it has widespread applications in various sectors such as food industries, bio-medical sectors, and for the formation of biobased polymers and nanocomposites [7,8,9].

For the past few decades, with the increasing awareness of environmental sustainability, there is an increasing surge for “going green”, which has started searching for new biobased materials with high performance at affordable costs. Apart from having extensive utilization in the food and food packaging sectors, BC has several applications in “green” composite materials [5]. However, due to its high biocompatibility and biodegradability and other unique intrinsic properties, it is easier to functionalize BC to introduce antibacterial functional groups. This natural hydrogel is found to have promising applications in wound healing, as dry wounds need added moisture to ensure tissue regeneration. Although few review reports are available on bacterial cellulose [10,11,12], most of them focus on the biomedical applications of this versatile fiber. Hence, a review report is required to emphasize the antimicrobial and antibiofilm activities of the bacterial cellulose and its composites.

Utilization and optimization of different nutrient media have been prioritized in BC production to curtail downstream costs, and its antibacterial activity has been enhanced by organic, inorganic, and polymeric compounds and nanoparticles [13,14].

The present overview discusses the bacterial production and unique features of bacterial cellulose with a special reference to its antimicrobial applications.

## 2. Properties of Bacterial Cellulose

Cellulose is the most liberal and viable sustainable polymer, and it is the one that is predominantly utilized. Though cellulose is considered a plant-based product, several fermentation techniques showed positive results in producing alternative sources of cellulose from bacterial genera. Though the BC and plant fiber have different physical and chemical properties, they have a similar structure with two discrete cellulosic subunits I and II [15]. The polymerization degree of cellulose varies from 2000–6000 in BC to 13,000–14,000 in plan cellulose. The 3D mesh-like structure of the cellulose prevents microbial attack. The *β*-1 → 4 glycosidic linkage is responsible for the free surface hydroxyl group in the surface area of the cellulose. BC is 10–50 nm in diameter with 100–1000 nm in length [16,17].

**Figure 1:**
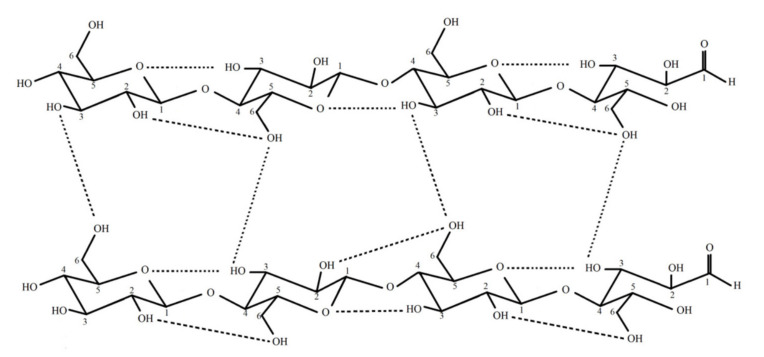
Inter and Intra hydrogen bonding between the bacterial cellulose (edited from Festucci-Buselli et al., 2007)) adapted from [21].

Hydrogen bonds are plentiful within cellulose (Figure 1) due to the presence of a large number of oxygen atoms and hydroxyl groups [18]. Parallel stacking is observed within cellulose due to the presence of van der Waals force that helps in the development of crystalline nanofibers followed by the development of microfibrillar structure [19]. The presence of the supercoil structure helps in promoting the hierarchical orders that help in providing a very high amount of mechanical strength to cellulose [20].

## 3. Bacterial Cellulose Producing Bacteria

Very few bacterial species can synthesize cellulose, among which is Gram-negative bacterium *Gluconacetobacter xylinus* (conventionally known as *Acetobacter xylinum*) [22]. Researchers discovered the bacterium responsible for the production of cellulose as microfibrils on a large scale [4,23]. This group of Gram-negative, rod-shaped aerobic bacteria, due to the high yield of cellulose, is considered to be a model organism for the production of BC [24] for commercial fermentation.

Apart from this group, BC can be synthesized by other bacterial genera such as *Agrobacterium* spp., *Acetobacter* spp., *Azotobacter, Rhizobium* spp., *Sarcina, Alcaligenes,* and *Pseudomonas* [25]. Acetobacter, a common vinegar bacterium, is a non-photosynthetic advanced purple bacteria that can convert glucose, glycerol, sugar, or any other organic substances into pure cellulose [26,27]. In the growth medium, microfibrils are merged from each synthetic site to form large-ribboned cellulose, which is non-motile due to the formation of floating pellicles with the entangled associated cells. Apart from plant host cells, tumor-forming bacterium *Agrobacterium tumefaciens* also secretes cellulose fibrils which surround the cell in order to promote cell attachment and virulence [28]. Both *Agrobacterium tumefaciens* and *Gluconacetobacter xylinus* can be grown widely in a suitable medium, whereas mutants of both species are impaired in cellulose biosynthesis. The genus *Komagataeibacter* has also been found to be noteworthy for its cellulose producing ability [29].

**Figure 2:**
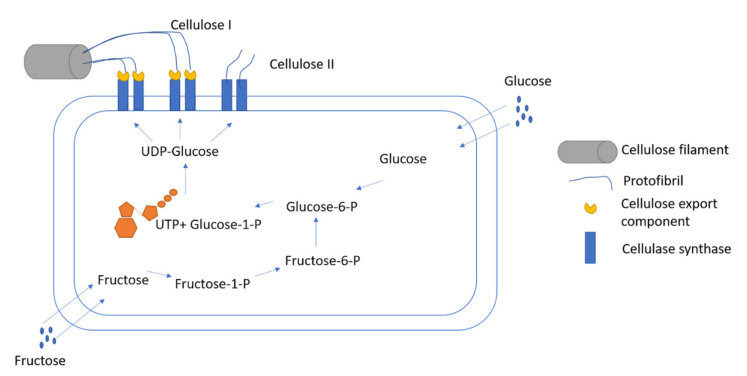
Biosynthesis of cellulose I and cellulose II from glucose and fructose in bacterial cell.

The production of cellulose take place under the presence of various types of precursors, including hexanoates, hexoses, the three carbon compounds pyruvate, dihydroxyacetone, pyruvate, and glycerol, along with the presence of four-carbon compounds such as dicarboxylic acid, which is associated with citrate cycle [30]. The rate of synthesis of the BC remains unaffected by the inhibitors that are associated with protein synthesis at the resting stage of the bacterial cells [31]. It has been observed that bacteria similar to *A. xylinum* retain the cytoplasmic enzymes when incubated with UDP-[14C] glucose in the presence of bis-(3′,5′)- cyclic diguanylic acid (c-di-GMP) or GTP. *A. xylinum* is the model organism in which the entire phenomenon of the development of BC can be observed from glucose. The mechanisms of phosphorylation of glucose in the presence of glucokinase followed by the process of isomerization of glucose-6-phosphate to glucose-1-phosphate in the presence of the enzyme phosphoglucomutase act as important steps in the synthesis of BC (Figure 2).

## 4. Cultivation of Bacteria for the Production of Bacterial Cellulose

Fermentation for the production of BC is conducted in static and agitated or stirred mode, and with the change of mode, different forms of cellulose are produced. Under the static condition, three-dimensional interconnected reticular pellicles are formed, whereas sharp, irregular sphere-like cellulose particles (SCP) are produced in agitated or stirred conditions. Cellulose formation under static conditions is regulated by the supply of carbon and air into the medium. BC formation is increased with the increase in growth time and the C-H bonding. When the pellicle growth slows down and all the bacteria are entrapped, the synthesis of BC reaches its threshold. Compared to continuous processes, semi continuous processes are put forward in all the industrial scale in order to achieve maximum BC productivity. For commercial production of BC with high yield, agitated fermentation has been used over static fermentation. 

The production of BC can be achieved by both agitated and static fermentation. The process involved in the production of BC depends on the morphologies and the properties of BC to be produced [32]. The formation of gelatinous pellicles takes place in static culture at the air–liquid interface of the culture media, whereas in an agitated fermentation system, the irregular pellets are developed and remain totally suspended in the culture media. Since higher genetic stability is found among the bacterial species, cultured by static fermentation technique [4], the agitated fermentation can be more easily scaled up for the purpose of industrial production [19], although there may be a chance of the appearance of non-cellulosic bacterial mutant that can drastically decrease the productivity [12]. Varied microscopic morphology with 3D reticulate network has been observed with the BC obtained by static or agitation-based fermentation mechanisms [22]. The BC obtained from the agitation fermentation possesses a very low degree of polymerization and also exhibits a lower level of crystallinity in comparison to those obtained from the static fermentation techniques [33]. CP/MAS 13C NMR analysis reveals that the proportion of Iα is lower but Iβ is quite higher in agitation fermentation obtained BC than that of BC yielded from static fermentation [34]. The mechanical properties vary in BC obtained from static to agitation fermentation, since Young’s modulus of the BC obtained from static fermentation technique exhibits a higher value in comparison to those obtained from the agitation fermentation technique [35]. It has been observed that BC produced by the technique of static fermentation requires raw materials possessing fixed geometrics, high water holding capacity, and good wet tensile strength. The optimized culture media required for production of BC include 0.5 wt% peptone, 0.5 wt% yeast extract, 0.27 wt% Na_2_HPO_4_, 2.0 wt% glucose, and 0.115 wt% citric acid [36]. The cost of production of BC is too high for it to be sustained for various industrial processes; thus, alternative strategies are being studied for the development of cost-effective mechanisms [37]. Various mechanisms involve promotion in the production of BC, including the isolation of bacterial strains that are responsible for the production of BC and the detection of high-yielding strains with the use of genetic engineering and traditional mutagenic methods [38] and optimization of the various culture conditions [35]. Various types of carbon sources such as sucrose, fructose, molasses, arabitol, and mannitol, and nitrogen sources such as peptone, yeast extract, and corn steep liquor, are used for the purpose of producing BC [39]. Various types of agricultural residues can be also used for the production of BC [40].

The mechanism of fermentation is followed by the removal of impure raw pellets of BC that comprise metabolic substances and nutrient residues along with BC. The mechanism of purification can be achieved by the treatment of BC with alkaline solution at a temperature of 1000 °C for 15–20 min to remove the bacterial cells. This is followed by the washing of the pellets with distilled water to recover the BC pellets and recover the value of pH [17].

Interestingly, a cell-free enzyme system is also developed to produce BC, which might transform into a cell-free factory for BC production in the future. The cell-free enzyme system is developed from BC-producing strains and contains whole enzymes and cofactors required for BC synthesis. Quantitative analysis reveals that the system produces BC with a higher yield than the corresponding bacteria [41]. Further study demonstrates that the cell-free enzyme system produces BC via an anaerobic biosynthesis process, and the premature BC pellicles formed in the culture media move to the air–liquid interface and assemble into a sheet [42]. Β-(1-4)-glucan chains become polymerized into the cell wall before being delivered into the culture medium. The mess structure of the BC gives it pores through which cellulose-synthesizing complex perceives place between plasma membrane and outer membrane of the cells. In this mechanism, the initial material, uridine diphosphate glucose (UDP glucose), is expanded into the cellulose chain, resulting in the development of basic fibril, which is assembled with the elementary fibrils in order to develop microfibrils and strips [43,44,45]. 

The fermentation medium is incubated for 1–14 days in pH 4–7, 28 to 30 °C with the inoculum, until the vessel gets filled by cellulose. The proper aeration and formation of CO_2_ control the metabolic activity in the production of BC. Compared to static cultivation, agitated cultivation is expensive due to the continuous agitation, which increases production yield [46]. In the stirred cultivation process, cellulose is produced in the form of solid balls. The increase in the shear rate may increase the bio productivity, although elevated share rate results in formation of turbulence force in the medium, leading to the change of cellulose-producing strains to the cellulose negative strains. Both the stirred tank bioreactor and air-lift bioreactor showed positive results with high productivity of the BC in highly viscous and dense fibrous suspensions. In the context of oxygen mixing, an air-lift bioreactor showed more efficiency over a stirred tank reactor, as from the bottom, the vessel oxygen is transferred continuously to the culture medium in order to provide an aerobic atmosphere. An airlift bioreactor showed efficiency in controlling energy and shear stress to control the production of cellulose-negative mutants. 

## 5. Parameters Controlling BC Production

Several parameters such as dissolved oxygen, pH, and temperature, independent of static or agitated cultivation, need to be optimized to improve BC yield (Table 1) [47]. Crystalline polymorph, crystallinity index (CI), cellulose Iα, and size are the factors that determine microstructure, and these are dependent on culture conditions [48]. 

### 5.1. Temperature

One of the most important parameters is the temperature, which can regulate the adaptation pattern of an organism for its survival by influencing the normal homeostatic physiology. A temperature range of 25 to 30 °C was found to be best for the production of BC, as a Komagataeibacter sp. Was cultivated at 30 °C for 7 days under static conditions [29], whereas 28 °C is the optimum temperature for the BC production by *Acetobacter xylinum* [27,49]. A slightly higher temperature of 33.5 °C was required by *Acetobacter senegalensis* MA1 [50]. For BC production by *Gluconacetobacter,* sp. RV28, *Pseudomonas* sp. RV14, and *Enterobacter* sp. RV11 preferred a range of temperature that was found to be 28–30 °C [51], as high temperatures cause denaturation of the culture environment, whereas low temperatures slow down cellular metabolism by supplying low energy for cell development.

### 5.2. pH

pH is another important factor in controlling oxidative fermentation of BC production. Acidic or near-neutral pH is suitable for BC production. During the fermentation process of BC, production of secondary metabolites such as acetic acid, gluconic acid, and lactic acid independently shifts the pH of fermentation culture media [47]. Thus, pH 4–6 is considered the ideal pH for the fermentation culture medium of BC. Experimental observations indicate that pH of 5.50 for *Acetobacter xylinum* [52], 4.5 to 7.5 for another strain of *Acetobacter xylinum* [27], and 6.0 for *Komagataeibacter* spp. [29] are required.

### 5.3. Culture Media

Carbon, in the form of fructose, glycerol, maltose, starch, and xylose, and nitrogen, in the form of casein hydrolysate and peptone, are the main components of the growth medium required for BC fermentation. Alteration of growth media has direct or indirect effects on microbial growth patterns. A 5 g/L yield of BC and a 4.8 g/L yield of BC from *A. xylinum* were reported in presence of casein hydrolysate and peptone, respectively [53]. Vitamins are important in regulating cellular metabolism and growth. Apart from pantothenate and riboflavin, vitamins such as pyridoxine, nicotinic acid, biotin, and p-aminobenzoic acid are required for cellular synthesis [54]. It was observed that in the presence of lignosulphonate, a low formation rate of gluconic acid increases the productivity of BC [55]. A medium with 0.38% agar, 2.85% corn steep liquor, 4.99% fructose, and 28.33% dissolved O2 is suitable for the formation of 14.0 g/L of BC [56]. Optimized cultivation of *Gluconacetobacter* sp. RKY5 in agitated and static culture produces 5.63 g/L and 4.59 g/L of BC, respectively [57]. Using dual carbon sources such as fructose and sucrose, around 8.79 g/L yield of BC was reported. Cheaper carbon sources such as molasses, muskmelon, orange juice, and watermelon can be used for BC production, and among them, muskmelon is recognized for the highest yield of BC 0.08 g/L. Even so, BC-producing cells have the unique feature of using the nutritional medium for the inexpensive production process. 

### 5.4. Agitation Rate

Production of BC is affected by another important parameter, i.e., agitation rate. At a lower agitation rate of 100 rpm, a uniform solid ball of 0.5 to 1 cm diameter is formed. With an increase of agitation rate around 150 to 250 rpm, BC size decreases, and at 300 rpm, irregularly shaped clumps are produced. Net 3, 10, 11.46, 7.73, and 3.91 g/L yields of BC were obtained at 100, 150, 250, and 300 rpm, respectively, in the case of synthesis of cellulose from *G. xylinus* DSM46602. According to this observation, the moderate rpm is suitable for the optimal production of BC. The generation of shear stress during agitation provides stability to the mutant of cellulose-producing strains in order to enhance the net BC productivity. An increase in the impeller speed from 80 to 500 rpm may decrease the cellulose-negative strains to almost zero. Agitation rate has an impact on the size of the BC. It may decrease the size of BC from 8 mm to <1 mm [65]. The agitation rate needs to be optimized for the production of BC on a large scale as it varies for different microbes along with the culture medium. 

### 5.5. Oxygen Level

Oxygen plays an important role in controlling aeration within the media. An adequate supply of oxygen is needed as all the microbes in the culture medium are aerobic in nature. Within the media, a low level of dissolved oxygen obstructs bacterial growth, leading to the retardation of BC production. O_2_ is essential for cellular metabolism and BC production. A restricted O_2_ supply not only collapses the BC production but also reduces BC quality. O_2_ transfer rate has an inverse relationship with the viscosity of the broth. An increase in viscosity decreases the O_2_ transfer rate as well as the BC productivity. Ten percent saturation of dissolved oxygen provides the highest yield of BC in the fed-batch cultivation [66]. Therefore, to obtain the highest yield of BC, a two-stage cultivation system has been developed by Liu et al., wherein in the first-stage dissolved O_2_ within the culture media is increased before reaching the log phase of the growth curve followed by maintaining the hypoxic condition at the secondary phase of the growth curve in the second phase for BC production [67].

### 5.6. Growth Curve

Analogously to the other bacterial strains, BC-producing strains have identical growth curve patterns with lag, log, stationary, and death phases. *Komagataeibacter mendellinensis* has a lag phase of 25 h when it is cultivated in growth media with carbon sources glucose, fructose, and sucrose in 2% *w/v* [68]. A five percent fructose in the culture medium is longer than the lag phase by around 10–15 h in the case of *G. liquefaciens*, leading to the long-time consumption in BC synthesis.

### 5.7. Yield of BC

Different bacterial strains favor different nutritional media independently of the cultivation technique. Cheap media such as citrus waste solution, pineapple peel, or molasses also assist in the production of BC. The 2 to 14 days range is optimal for regulating cultivation of BC in order to obtain the highest yield. Overall increase in incubation days may increase the yield of BC. Among all other sources, glucose is the main nutrient source of carbon, which is remarkably consumed by bacterial cells to attain the metabolic demands and high energy yield. 

## 6. Bacterial Cellulose Biocomposites and Their Characterization

Bacterial cellulose has eccentric mechanical, morphological, and structural properties (Table 2). Due to these characteristics, BC has applications in diverse sectors including wound dressing, paper restoration, and blood vessel regeneration. In spite of having special characteristics, BC has restricted applications due to a lack of antibacterial properties, optical transparency, and stress-bearing capability. In order to overcome these limitations, BC-based composites containing matrix and reinforcement material were introduced. The porous arrangement of fibers in addition to reinforcement materials make the matrix a biocomposite that has biological and physicochemical properties. BC composites are synthesized using an in situ method, where reinforcement materials are added into it during synthesis, and an ex situ method, where BCs are infused with reinforcement materials.

## 7. Antimicrobial Activities of BC Composites

### 7.1. Deposition of BC on Electrospun PLA

In Situ deposition of BC on electrospun PLA was illustrated by Xiang and Acevedo in 2017. In 8 wt% in chloroform/acetone (3:1 v/v) solution, PLA was dissolved followed by the acquisition of PLA fiber mats with 100 mm cross-sectional diameter through electrospinning. BC/PLA spectral bands within the nanocomposites showed absorption peaks at 1746 cm^−1^ and 3200–3500 cm^−1^, and they showed O-H stretching bands due to the modification of electrospun PLA with BC nanofibers [79]. Incorporation of PLA in BC does not influence the crystalline morphology of BC, although a higher CI of 83.3% ± 4.3% was derived from the XRD analysis of BC/PLA composites [79]. Electrospun nanofibers containing BC functionalized with antimicrobial agents have shown enhanced antibacterial performance compared to traditional dressings [80] and are nowadays regarded as a promising wound dressing material due to their antibacterial, antiviral, and anti-inflammatory activities, as well as their ability to maintain an appropriate environment for wound healing [81].

### 7.2. BC-Reinforced Fabrics with Natural Fibers

In order to produce hierarchical fiber-reinforced nanocomposites, BC is cultivated on hemp and sisal fiber via agitation [82]. In order to prepare green nanocomposite, BC is bonded with flax fabric (FF) and little PLA was used to wet the FF attached BC through the casting method. BC-modified hemp fiber is arbitrarily organized, having a diameter of 50–100 nm, and it encircles the fiber surface [83]. The pore size between the two fabric threads is reduced, resulting in the uniform distribution of PLA-modified BC-attached FF composites (PLA–BC–FF). Antibacterial activity is manifested by the PLA–BC–FF biocomposites, which was revealed by forming zone of inhibition against Gram-negative *E.coli*.

Antibacterial activity of the BC composites’ surface functionalized with benzalkonium chloride-based product was found against *Escherichia coli* and *Staphylococcus aureus* [84].

### 7.3. BC-Reinforced Chitosan (BC-Ch) Composite

The specific two methods i.e., impregnation by immersion and impregnation in mass, are mainly used for preparation of BC-Ch composite. The whole BC is immersed directly into the chitosan molecules in the form of paper or matrix, and then it proceeds to dry in the case of BC-ChI. For preparing BC-ChM BC is mixed to chitosan mechanically and incubated for a fixed time [85]. Besides these two methods, another method is used for BC-Ch preparation wherein chitosan is placed inside the bioreactor, and in each step, 0.25% of chitosan concentration is incremented from 0 to 0.75% [86]. Chitosan is uniformly distributed throughout the BC-Ch nanocomposite, and immersing BC within the chitosan molecules enhances the binding and interaction of the surface fibers. BC-ChI has more condensed morphology than BC-ChM [87]. BC-Ch nanocomposite fiber size is asymmetric due to the impediment of the assembly of BC nanofibers. Impregnation of chitosan into natural BC showed a reduction in the bacterial growth upon the fibers. The antibacterial assay provides the result of the consequential diminution of Gram-positive *Staphylococcus aureus* and Gram-negative *Pseudomonas aeruginosa*. It was also observed that BC-ChM is more effective compared to BC-ChI in inhibiting microbial growth due to its firm surface and bacteriostatic activity of the chitosan [86]. The BC/Ch composite showed antibacterial activity against *S. aureus, E. coli,* and *P. aeruginosa* [88]. BC-Ch nanocomposites are also found to have antimicrobial effect against the yeast *Candida albicans* [89].

Chitosan/bacterial cellulose composite films containing diamond nanoparticles (NDs) were found to be effective against *Staphylococcus aureus* ATCC 25,922 and *Escherichia coli* ATCC) 25,923 [90]. The antibacterial activity of the starch/chitosan-based biocomposite films with BC was assayed found against *Staphylococcus aureus*, *Bacillus subtilis*, *Escherichia coli,* and *Pseudomonas aeruginosa* [91].

### 7.4. Montmorillonite (MMT)-Reinforced BC Composite

Suspension culture and in situ preparation are the two main methods used to prepare BC biocomposites reinforced with MMT (BCMMT). Different concentrations of MMT (0.5, 1.0, 2.0%) are mixed with BC by a mechanical shaker for 72 h at 150 rpm to obtain BCMMT0.5, BCMMT1, and BCMMT2. Similar concentration of MMT is added to the water within the ultrasonicator for 1 h to make MMT/water dispersion followed by addition of it into the BC growing medium for 13 days at 28 °C in order to produce in situ BCMMT composite [92]. In Situ BCMMT biocomposite has a compact network with MMT nanolayers and BC nanofibers [63]. The antibacterial activities of the composites were then assessed against *Escherichia coli* and *Staphylococcus aureus* through the disc diffusion assay. Such antibacterial activities are utilized in the development of BC sheets as wound dressings and regeneration materials for therapeutic applications without any side effects [93].

## 8. BC-CU NPs Nanocomposites

The combination of copper nanostructure along with BC showed an effective antimicrobial activity. It has been observed that the nanocomposite showed antibacterial potential against nosocomial organisms such as *Klebsiella pneumonia* and *Staphylococcus aureus*. The chemical structure and morphology of the nanocomposite provided a great efficacy to act as potent antibacterial agent. The BC composite showed an effective antibacterial potential along with the nanocomposite [94].

## 9. BC–Graphene-CuO Nanocomposites

The antibacterial potential of the BC-graphene CUO showed marked antibacterial potential. It has been observed the CuO nanosheets have a length of 50–200 nm and a width of 20–50 nm. The nanocomposites exhibited its activity against Gram-positive bacteria in comparison to that of the Gram-negative bacteria. It has been further observed that the BC-grapheneoxide-CuO nanocomposites have greater efficacy in comparison to that of the BC-CuO nanocomposites. It further exhibited biocompatibility with that of the mice fibroblast cells and can be used as an excellent antibacterial material [95].

## 10. BC–ZnO Nanocomposites

BC-ZnO nanocomposites showed excellent antibacterial potential against Gram-positive and Gram-negative bacterial cells and had excellent biomaterial properties. They also exhibited photocatalytic activity and were able to degrade 91% methyl orange dye under the influence of UV irradiation for a period of 2 h [96].

## 11. BC–Methylglyoxal Nanocomposites

BC cellulose produced by *Acetobacter xylinum* exhibited wound-healing properties in association with methylglyoxal, resulting in the formation of BC-Methylglyoxal nanocomposites. They exhibited stability at a higher range of temperature and exhibited a considerable amount of mechanical strength. The nanocomposite showed its efficacy against *Staphylococcus aureus, Micrococcus luteus, Pseudomonas aeruginosa,* and *Escherichia coli,* thereby making it an alternative would-healing biocompatible material [97].

## 12. Antimicrobial Activities of Bacterial Cellulose Augmented with Other Compounds

Bacterial cellulose supplemented with povidone-iodine, polyhexamethylene biguanide, and octenidine-like antimicrobial agents showed around 1.5-fold increased antimicrobial activities against both Gram-positive and Gram-negative bacteria. The overall compressive strength of the combined material was augmented, increasing the antimicrobial activity by around 65% against *S. aureus* [98]. A cationic surfactant benzalkonium chloride exhibited antimicrobial activities against Gram-positive bacteria and can also be used for wound dressing. After being submerged for 24 h, EDA-DLA-Tyr becomes attached to bacterial cellulose, and the composite shows antibacterial activity against *Staphylococcus aureus* and *Staphylococcus epidermidis*. However, the activity of the bacterial cellulose is enhanced when the antibiotic amoxicillin is incorporated into it. In order to make the formation of cross-linkage between BC and amoxicillin, BC is treated with 3-aminopropyl-trieoxysilane to create the bond with aminoalkysilane groups through Si-O-C bonding. After treatment with the drug carbodiimide-crosslinked EDC/NHS (1-ethyl-3-(3-dimethyl aminopropyl)carbodiimide hydrochloride), the carboxylic group is added to amoxicillin. The modified amoxicillin with the activated ester group form covalent bonds to the NH2 of BC, resulting in the formation of a BC composite that is able to reduce 95% of the *C. albicans, E. coli,* and *S. aureus* cell viability [99]. Eighty-two percent of the all loaded drugs are released within 48 h application of the tetracycline-loaded BC, and it is able to reduce 99.9% of the viability of the *S. aureus* and *B. subtilis* [100].

## 13. Antibiofilm Activities of BC Composites

### 13.1. Antibiofilm Effect of Bacterial Cellulose-Tannic Acid Composite

Biofilm refers to a consortium of microorganisms that remain embedded within the self-produced extracellular polymeric substances (EPS) upon the biotic or abiotic inert surface [101]. EPS includes exopolysaccharides, proteins, and extracellular DNA, which manifest the strong barrier against any external stresses such as antibiotics or host immune responses [102]. Biofilm is responsible for 80% of the microbial infections associated with bacteria, fungi, protozoa, viruses, or bacteriophages in the human body [103]. Complete lavage of infections becomes difficult when biofilm is formed upon the wound bed, even though a small portion of bacterial cells remains attached to the wound bed after treatment with the strong antibiotics, resulting in regeneration of biofilm with high infections [104]. Apart from the growth upon abiotic surfaces of medical devices such cardiac pacemakers, contact lenses, urinary and intravascular catheters, and tissue fillings, it is responsible for about 60–70% of the nosocomial infections. To combat the biofilm-mediated infections, the main strategy is to inhibit bacterial adhesion and its growth, which may not be achieved by using only antibiotics, since biofilm bacteria are impenetrable and difficult to remove, leading to the emergence of antibiotic resistance. The development of antifouling or antimicrobial surfaces can be an alternative strategy to arrest microbial adhesion. Another therapeutic strategy could be using microbial peptides against biofilm formation. Photodynamic therapy can be one of the potential antibiofilm methods with several side effects such as residual skin photosensitivity and pain in the treated area. Quorum quenching and inhibition of intra- or inter-bacterial signaling are the main strategies to inhibit biofilm formation, which can be accomplished by tannic acid, and hence are used against two chronic infectious pathogens *S. aureus* and *P. aeruginosa* [105,106].

Since TA shows antibacterial activity against a wide range of invasive bacteria causing wound infections, and since 3D composites made of TA-graphene show antibacterial activity [107], TA is effectively used to make composites with BC. BC, as a natural biopolymer with high mechanical strength, water uptake, and biocompatibility, has 3D-nanofiber networks and chemical reactive groups, which facilitate the penetration of molecules into its inner space. Incorporation of TA into BC creates a shortage of microbial activity of the BC. Magnesium acts as the crosslinker between the TA and the BC in order to form BC-TA-Mg biocomposite. The novel BC-TA-Mg composite exhibits antimicrobial and antibiofilm activity and shows antimicrobial activity against *S. aureus, P. aeruginosa,* and *E. coli* with the zone of inhibition of 17.30 ± 0.53 mm, 15.07 ± 1.05 mm, and 14.39 ± 0.80 mm diameter, respectively. Diameter of inhibition also shows that BC-TA-Mg is more effective on Gram-negative bacteria compared to Gram-positive. Since BC does not have any negative impact on biofilm formation, preparation of BC-TA-Mg biocomposite is required to inhibit biofilm formation. It was found that 24 h incubation with composite could effectively reduce the viable cell count of *S. aureus* and *P. aeruginosa*. Although Mg^2+^ affects the inhibitory effect of the composite by stimulating cell–cell adhesion and aggregation by interacting with cell-wall teichoic acid, it does not hamper the anti-biofilm activity against *S. aureus* and *P. aeruginosa*. 

### 13.2. Antibiofilm Effect of Silver–BC Composite

Another method of enhancing the antibacterial activity is the incorporation of silver particles to produce BC–silver particle composites. Silver shows a robust cytotoxicity against a broad spectrum of micro-organisms. Due to the presence of an oligodynamic effect, silver ions exhibit bacteriostatic as well as bactericidal effects. Silver nanoparticles containing BC membranes can decompose silver nitrate hydrolytically with the help of triethanolamine complex. TEA is used as stabilizer as well as a reducing agent, leading to the dispersion of spherical particles [108]. BC-Ag-TEA composites show strong antimicrobial activity against both Gram-positive and Gram-negative bacteria. A 1 mol/L concentration of BC-Ag-TEA composite shows activity against *Staphylococcus aureus* and *Pseudomonas aeruginosa*. A 2 cm zone of inhibition in agar diffusion method allowed the diffusion of silver NP in culture medium from BC-Ag-TEA composite [109]. Silver ions interact with biological macromolecules via the thiol (-SH) group of proteins and replace the H+ of the sulfhydryl or thiol groups by Ag+, resulting in the inactivation of proteins and the decrease of cellular permeability, leading to cell death. Upon the bacterial cell surface, a stable -S-Ag group is produced via the reaction between the monovalent silver and the sulfhydryl group. Silver ions are incapable of penetrating the cell membranes and do not react with interior -S-H groups, resulting in persistence of silver in nontoxic form [110].

### 13.3. Antibiofilm Activity of Chitosan–BC Composite

**Figure 3:**
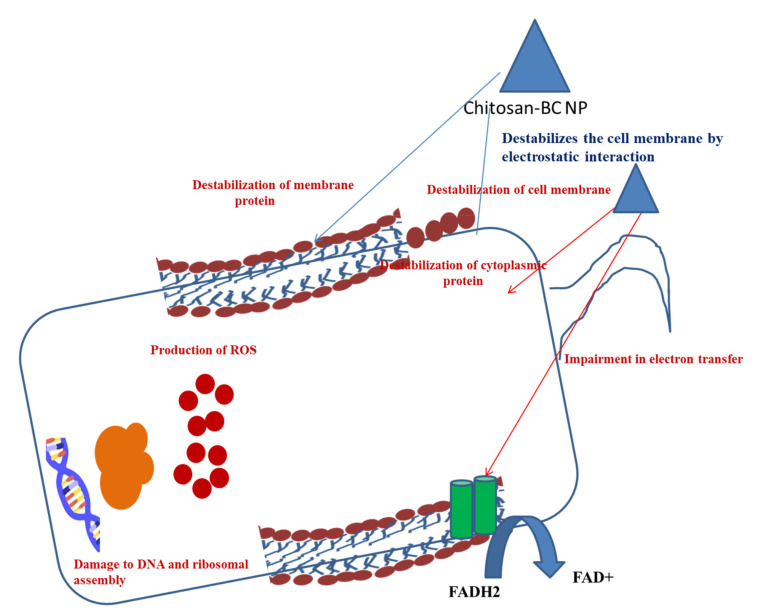
Antimicrobial effects of chitosan BC composite.

Chitosan is considered an antibacterial agent as it is able to bind to the negatively charged bacterial cell wall, leading to the alteration of cell permeability and cell disruption (Figure 3). It also becomes attached to the DNA of the cells, resulting in the inhibition of DNA replication and cell apoptosis [111]. Furthermore, chitosan acts as a chelating agent, which binds to the metal elements of the cell and exhibits toxin production, resulting in inhibition of microbial growth [112]. The polycationic structure of the chitosan is responsible for the antibacterial activity [113,114]. The pH below the pKa of the chitosan-based BC composite helps the electrostatic interactions between the composite and the anionic components of the microorganisms. This is the principle of the antibacterial activity of the chitosan-based BC composite [115]. A higher pH condition is responsible for the protonation of chitosan, resulting in an increase in the antibacterial properties. Apart from protonation, the linkage between the amino groups and C-2 on chitosan backbones is required for electrostatic interactions. The incorporation of amino groups enhances antimicrobial activity of the composite. Asparagine N-conjugated chitosan oligosaccharide has two positively charged sites, which allow stable interaction with carboxyl-negative charges on the bacterial cell wall [116]. Chitosan, along with nanoparticles, alter cell permeability by interacting with the cell surface and interrupting the entry of the essential solute into the cells. 

Incorporation of nanomaterial into the chitosan reveals the anti-biofilm properties against both the Gram-positive *Listeria monocytogenes* and *S. aureus* and Gram-negative *P. aeruginosa* and *S. typhimurium*. Chitosan–streptomycin gold nanoparticles are able to disrupt the EPS of the biofilms in order to inhibit biofilm formation. Streptomycin conjugated chitosan-gold nanomaterial can penetrate through the biofilm matrix and develop contact with the bacterial surface, leading to the enhancement of the bactericidal effect. Methylene blue (MB)-mediated antimicrobial photodynamic inactivation (APDI) is enhanced by the chitosan nanomaterial and stimulates disruption of *S. aureus* and *P. aeruginosa* biofilms [117].

Apart from these three BC composites, several active compounds are amalgamated with BC to enhance its activity against microbes. Polyhexanide, octenidine dihydrochloride, benzalkonium chloride, tetracycline, amoxicillin, povidone iodine, lysozyme, dehydrogenative polymers, zinc oxide, and gold nanoparticles are the few compounds that are reported to be amalgamated with the BC. All of these composites possess antibacterial and antibiofilm activities. 

## 14. Dye- and Heavy-Metal-Removal Activities of BC Composites

**Figure 4:**
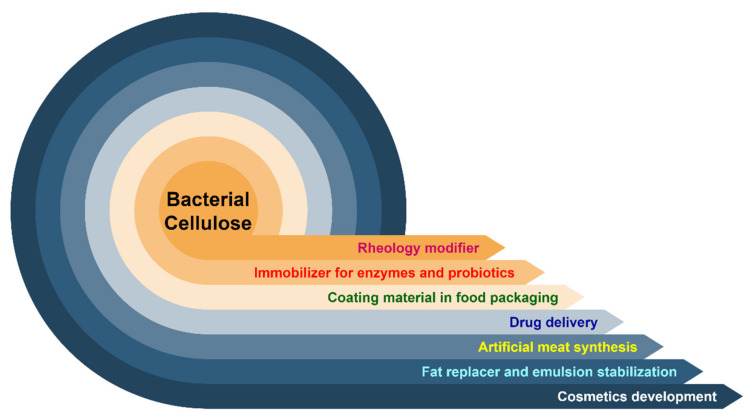
Applications of bacterial cellulose in different fields.

Heavy metals such as Pb^2+^, Cu^2+^, and Cr^6+^, and anionic organic dyes such as Congo red (CR) can be removed by batch adsorption by bc-attapulgite magnetic composites [118]. In another experiment, Pb^2+^ and Cu^2+^ could be successfully adsorbed on composite hydrogel of BC prior to removal [119]. Figure 4 represents various applications of BC.

## 15. Conclusions

Bacterial cellulose, a versatile biopolymer with antimicrobial and antibiofilm efficacy, can be successfully used in therapeutic approaches. Besides its biocompatibility and biodegradability, it has numerous advantages over plant cellulose, such as purity, high water-uptake and -holding capacity, high crystallinity, high porosity and permeability to gas and liquid, and high tensile strength with mechanical robustness, which has made this natural polymer an excellent medium to be used for wound dressings, composites, dental grafts, and gels. This pure form of cellulose has zero level of toxicity and no side effects. BC biocomposites’ antibiofilm efficacy has opened up a new dimension in treating biofilm-mediated chronic wounds and infections, although the only constraint is the limited productivity of BC. The special drive is to be taken for scaling up in the production of BC by designing new fermenters, enduring mechanical agitation of the growing microorganisms and adopting newer biotechnological approaches to curtail the production cost by using cheap sources such as agricultural wastes or industry byproducts. Mass production of bacterial cellulose in the future will lead to the availability of a vital biomaterial that can be safely used for the production of biomedical devices, due to not only its mechanical uniqueness but also its versatile antimicrobial and antibiofilm efficacy.

## Figures and Tables

**Table 1:** Yield of BC with the variation of bacterial strains, culture medium, and cultivation methods.

Medium	Bacterial Strain	Incubation Days	Yield of BC g/L	Reference
Glycerol	*Gluconacetobacter* sp. *RKY5*	6	4.59 a 5.63 b	[57]
Glucose yeast extract broth	*Acetobacter xylinum K086*	7 days	0.14–0.39 a	[58]
	*Acetobacter xylinum K975*		1.11–1.55 a	
	*Acetobacter xylinum K428*		0.09–0.22 a	
	*Acetobacter xylinum K1011*		0.57–1.46 a	
	*Acetobacter xylinum KX*		1.14–1.84 a	
Glycerol	*Acetobacter* sp. *V6*	7 days	4.98 b	[59]
Molasse	*Komagataeibacter sucrofernentans H110* *Komagataeibacter hansenii C110*	14 days	8.2 ± 0.2 a, 8.1 ± 0.2 a	[60]
Stillage			9.5 ± 0.1 a, 9.2 ± 0.1 a	
Citrus waste solution	*Gluconacetobacter intermedius CIs26*	8 days	7.2 a	[61]
HS media			2.1 a	
Citrus waste modified HS			5.7 a	
Glucose	*Gluconacetobacter hansenii*	2	1.33 b	[62]
Glucose (modified HS Media)		14	14.72 a	[63]
Mannitol (modified HS media)		20		
Pineapple peel juice	*Gluconacetobacterswingsii*	13	2.8 a	[64]

a: static cultivation, b: agitated cultivation.

**Table 2:** Characterization of BC and its composites.

Characterization	Properties		References
	Absorption Peak	Functional Groups	
FTIR	~1160; ~1361; ~2895; ~3338	C–O–C antisymmetricbridge stretching of 1,4-β-d-glucoside; C–H bending; C–H stretching of CH2 and CH3; –OH stretching	[69]
	~1314; ~1426	CH2 groups out-of-plane bending; O–H in-plane bending	[70]
	~1108	C–C bonds of the monomer units of polysaccharide	[71]
	~1335	C–H deformation/–OH in-plane bending	[64]
	~900	Antisymmetric out-of-phase ring stretching for β-glucosidic linkages	[72]
	~1054	Bending of the C–O–H bond of carbohydrate	[73]
XRD	14.5°, 16.4°, and 22.5° diffraction peak corresponds to crystallographic planes of 101 (amorphous) and 200 (crystalline)		[74]
	Due to the presence of preferential parallel orientation of the cellulose fibrils, 1 12 crystallographic planes are missed in BC diffraction pattern		[75]
	intra and intermolecular H-bonding influences Crystallization		[76]
Tensile strength (TS) and Youngs’ modulus (YM)	0.26 ± 0.02 MPa TS and 0.005 ± 0.0003 MPa YM for Wet BC		[77]
	11.94 ± 1.15 MPa TS and 6.65 ± 0.16 MPa YM for lyophilized BC		
Scannining Electron Microscopy (SEM)	Helps in providing the structural morphology of the BC		[78]
Differential scanning calorimetry (DSC)	Helps in the determination of the stability of the BC		[78]
Viscosity measurements of the film-forming solution (FFS)	Helps in the purpose of analyzying the viscosity of the BC		[78]
Moisture content (MC)	Helps in the estimation of the water resistance capasity of the film		[78]
Water vapor permeability (WVP)			[78]
